# Systemic Inflammation Response Index Is a Predictor of Poor Survival in Locally Advanced Nasopharyngeal Carcinoma: A Propensity Score Matching Study

**DOI:** 10.3389/fonc.2020.575417

**Published:** 2020-12-10

**Authors:** Yuhua Feng, Na Zhang, Sisi Wang, Wen Zou, Yan He, Jin-an Ma, Ping Liu, Xianling Liu, Chunhong Hu, Tao Hou

**Affiliations:** Department of Oncology, The Second Xiangya Hospital, Central South University, Changsha, China

**Keywords:** nasopharyngeal carcinoma, locally advanced, prognosis, survival, systemic inflammation response index

## Abstract

**Introduction:**

Nasopharyngeal carcinoma (NPC) is a common malignancy in China and known prognostic factors are limited. In this study, neutrophil-to-lymphocyte ratio (NLR), platelet-to-lymphocyte ratio (PLR), systemic immune inflammation index (SII), and systemic inflammation response index (SIRI) were evaluated as prognostic factors in locally advanced NPC patients.

**Materials and Methods:**

NPC patients who received curative radiation or chemoradiation between January 2012 and December 2015 at the Second Xiangya Hospital were retrospectively reviewed, and a total of 516 patients were shortlisted. After propensity score matching (PSM), 417 patients were eventually enrolled. Laboratory and clinical data were collected from the patients’ records. Receiver operating characteristic curve analysis was used to determine the optimal cut-off value. Survival curves were analyzed using the Kaplan-Meier method. The Cox proportional hazard model was used to identify prognostic variables.

**Results:**

After PSM, all basic characteristics between patients in the high SIRI group and low SIRI group were balanced except for sex (*p*=0.001) and clinical stage (*p*=0.036). Univariate analysis showed that NLR (*p*=0.001), PLR (*p*=0.008), SII (*p*=0.001), and SIRI *(p*<0.001) were prognostic factors for progression-free survival (PFS) and overall survival (OS). However, further multivariate Cox regression analysis showed that only SIRI was an independent predictor of PFS and OS (hazard ratio (HR):2.83; 95% confidence interval (CI): 1.561-5.131; *p*=0.001, HR: 5.19; 95% CI: 2.588-10.406; *p*<0.001), respectively.

**Conclusion:**

Our findings indicate that SIRI might be a promising predictive indicator of locally advanced NPC patients.

## Introduction

Nasopharyngeal carcinoma (NPC) is a malignancy arising from the epithelium of the nasopharynx with a high incidence in endemic regions such as Southern China and Southeast Asia (1). More than 60% of patients are diagnosed at a locally advanced stage due to the lack of early symptoms and appropriate screening tools, and the prognosis remains poor (2). Induction chemotherapy combined with concurrent chemoradiation is the preferred treatment modality for locally advanced NPC (3). However, approximately 14% of patients relapse and 21% report metastasis (4). However, there is a persisting lack of prognostic biomarkers, and exploring prognostic factors is required for better patient stratification and treatment optimization.

There is increasing evidence that demonstrates inflammation to be one of the hallmarks of cancer, and it is closely related to cancer development (5). Activated inflammatory cells can promote cancer progression by inducing DNA damage or interfering with DNA repair systems (6). Recently, an increasing number of studies have demonstrated the value of inflammatory response biomarkers as predictive markers for prognosis in cancers. It has been shown that the neutrophil-to-lymphocyte ratio (NLR), platelet-to-lymphocyte ratio (PLR), and systemic immune inflammation index (SII) can predict the prognosis in various types of cancers (7–9). The systemic inflammation response index (SIRI) is a new systemic inflammatory response biomarker based on peripheral blood cells counts, and it was demonstrated to be effective in predicting the prognosis of esophagogastric junction adenocarcinoma (10), esophageal cancer (11) and cervical cancer (12). There is minimal evidence of SIRI as a prognostic marker in NPC from a previous study (13).

In this study, we aimed to evaluate the prognostic value of NLR, PLR, SII, and SIRI in a retrospective cohort of 417 locally advanced NPC patients.

## Materials and Methods

### Patient Selection

A cohort of patients diagnosed with NPC at the Second Xiangya Hospital, Central South University, from January 2012 to December 2015 was retrospectively analyzed. The inclusion criteria were as follows: (1) diagnosis of pathologically proven poorly differentiated nasopharyngeal squamous cell carcinoma, (2) stage III-IVa according to the 8th edition of the American Joint Committee on Cancer (AJCC) Staging System for NPC, (3) receiving radiotherapy or chemoradiotherapy in the Department of Oncology, the Second Xiangya Hospital, Central South University, with complete follow-up data available. The exclusion criteria were as follows: (1) history of chronic inflammatory diseases such as inflammatory bowel disease, (2) history of autoimmune diseases, and (3) acute infectious diseases within 4 weeks. All patients were followed up after completion of treatment at a pre-defined frequency of once every 3 months in the first 2 years, once every 6 months from the third to the fifth year, and once yearly thereafter. The last follow-up date was April 1, 2020. Relapse or metastasis of the disease was diagnosed on contrast-enhanced magnetic resonance imaging or computed tomography.

A total of 516 patients with NPC were shortlisted. To reduce patient selection bias, we used the propensity score matching (PSM) technique (14). PSM was developed using age, sex, stage, treatment mode, chemotherapy agent dose, chemotherapy agent type, etc. After PSM, 417 patients were finally included in this study. This study was conducted according to the Helsinki Declaration of 1975, revised in 2008, and approved by the ethics committee of the Second Xiangya Hospital, Central South University. And the written consent was waivered.

### Data Collection

Patients’ clinical information, including that on age, sex, Eastern Cooperative Oncology Group performance status (ECOG PS) scores, T stage, N stage, clinical stage, treatment mode, chemotherapy agent dose, and chemotherapy agent type, was collected, and routine blood results within 1 week before therapy were also collected. The NLR, PLR, SII, and SIRI were calculated using the following formula: NLR = neutrophil count/lymphocyte count, PLR = platelet count/lymphocyte count, SII = platelet count × neutrophil count/lymphocyte count, SIRI = neutrophil count × monocyte count/lymphocyte count. Overall survival (OS) was calculated from the date of diagnosis to the date of death due to any cause or to the date of the last follow-up; progression-free survival (PFS) was calculated from the date of diagnosis to the date of disease progression or death.

### Statistical Analysis

SPSS was used for data analysis (version 22.0; SPSS Inc., Chicago, IL, USA). Chi-square test was used to analyze the relationship between NLR, PLR, SII, SIRI, and clinicopathological features in locally advanced NPC patients. Receiver operating characteristic (ROC) curves were used to calculate the cut-off values for NLR, PLR, SII, and SIRI. When applying the ROC curves, the optimal cut-off value was defined as the value with the maximum Youden index (15). The Kaplan–Meier method was used to calculate the survival curves. Multivariate Cox hazard regression analysis was performed on the factors that were found to be significant in the univariate analysis. A two-sided *p* value of <0.05 was considered statistically significant.

## Results

### Patient Characteristics

The clinicopathological features of the 417 locally advanced NPC patients are shown in **Table 1**. The median age was 47 years (range: 14–81 years). A total of 321 (77%) were male and 96 (23%) were female. Most patients had an ECOG score of 0 to 1 (396, 99.3%). As for the T stage, 42 (10.1%) patients were T1, 135 (32.4%) were T2, 159 (38.1%) were T3, and 81 (19.4%) were T4. For the N stage, 17 (4.1%) patients had N0, 35 (8.4%) N1, 301 (72.2%) N2, and 64 (15.3%) N3. Overall, 282 (67.6%) patients were stage III, and 135 (32.4%) were stage IVa. A total of 124 (29.7%) patients received concurrent chemoradiation, and 293 (70.3%) received sequential chemoradiation. The total dosage of cisplatin was calculated as 266 (63.8%) patients with a dosage of ≥300 mg/m^2^ and 151 (36.2%) with a dosage of <300 mg/m^2^. Among all the patients, 273 (65.5%) received cisplatin-based chemotherapy, 131 (31.4%) received nedaplatin-based chemotherapy, while 13 (3.1%) received other platin-based chemotherapy.

**Table 1:** Clinicopathological characteristics of patients (n=417).

Characteristics	Number (%)
Age (years)	
Median	47
Range	14-81
Sex	
Male	321(77%)
Female	96(23%)
ECOG PS	
0	357(89.9%)
1	39(9.4%)
2	3(0.7%)
T stage	
1	42(10.1%)
2	135(32.4%)
3	159(38.1%)
4	81(19.4%)
N stage	
0	17(4.1%)
1	35(8.4%)
2	301(72.2%)
3	64(15.3%)
Clinical stage	
III	282(67.6%)
IVa	72(17.3%)
IVb	63(15.1%)
HGB (M/F) (g/L)	
<120/110	37(8.9%)
≥120/110	380(91.1%)
ALB (g/L)	
<43	255(61.1%)
≥43	162(38.9%)
Treatment mode	
Concurrent chemoradiation	124(29.7%)
Sequential chemoradiation	293(70.3%)
Total platin dose	
≥300 mg/m^2^	266(63.8%)
<300 mg/m^2^	151(36.2%)
Chemotherapy agent type	
Cisplatin	273(65.5%)
Nedaplatin	131(31.4%)
others	13(3.1%)

ECOG PS, Eastern Cooperative Oncology Group performance status; T, tumor; N, nodal; HGB, hemoglobin; M, male; F, female; ALB, albumin.

### Cut-Off Values of NLR, PLR, SII, and SIRI, and Their Correlation With Clinicopathological Features

**Figure 1:**
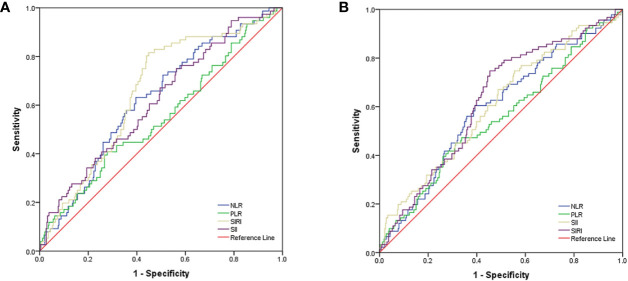
**(A)** ROC curve analysis for optimal cut-off value of NLR, PLR, SII and SIRI for PFS. **(B)** ROC curve analysis for optimal cut-off value of NLR, PLR, SII and SIRI for OS. ROC, receiver operating characteristic; PFS, progression free survival; OS, overall survival; NLR, neutrophil-to-lymphocyte ratio; PLR, platelet-to-lymphocyte ratio; SII, systemic immune inflammation index; SIRI, systemic inflammation response index.

As shown in **Figure 1A**, the area under the curves (AUC) of NLR, PLR, SII, and SIRI were 0.623, 0.548, 0.616, and 0.656, respectively, and the optimal cut-off values were 2.50, 163.06, 488.90, and 0.86, respectively. As shown in **Figure 1B**, for OS, the AUCs of NLR, PLR, SII, and SIRI were 0.592, 0.552, 0.605, and 0.625, respectively, and the optimal cut-off values were 2.50, 154.04, 488.90, and 0.86, respectively.

### Association Between NLR, PLR, SII, SIRI, and Clinicopathological Features

The association between NLR, PLR, SII, SIRI, and clinicopathological features in locally advanced NPC patients is shown in **Table 2**. A high PLR correlated with sex (p=0.001), and clinical stage (p=0.036). However, NLR, PLR, SII, and SIRI were not significantly correlated with patient age, ECOG PS, T stage, N stage, treatment mode, chemotherapy agent dose, or chemotherapy agent type (p>0.05).

**Table 2:** Clinicopathological characteristics according to NLR, PLR, SII, and SIRI.

Characteristics	NLR high	NLR low	*p*	PLR high	PLR low	*p*	SII high	SII low	*p*	SIRI high	SIRI low	*p*
Age (years)												
<65	174(41.7%)	228(54.7%)	0.797	124(29.7%)	278(66.7%)	0.250	241(57.8%)	161(38.6%)	0.604	201(48.2)	201(48.2%)	0.293
≥65	7(1.7%)	8(1.9%)		2(0.5%)	13(3.1%)		8(1.9%)	7(1.7%)		10(2.4%)	5(1.2%)	
Sex												
Male	135(32.4%)	186(44.6%)	0.348	84(20.1%)	237(56.8%)	**0.001**	185(44.4%)	136(32.6%)	0.124	164(39.3%)	157(37.6%)	0.728
Female	46(11.0%)	50(12.0%)		42(10.1%)	54(12.9%)		64(15.3%)	32(7.7%)		47(11.3%)	49(11.8%)	
ECOG PS												
0	158(38.1%)	216(51.8%)	0.366	115(27.6%)	260(62.4%)	0.494	223(53.5%)	152(36.5%)	0.552	188(45.1%)	187(44.8%)	0.776
1	21(5.0%)	18(4.3%)		11(2.6%)	28(6.7%)		25(6.0%)	14(3.4%)		21(5%)	18(4.3%)	
2	1(0.2%)	2(0.5%)		0(0%)	3(0.7%)		1(0.2%)	2(0.5%)		2(0.2%)	1(0.2%)	
T stage												
1	21(5.0%)	21(5.0%)	0.224	11(2.6%)	31(7.4%)	0.560	25(6.0%)	17(4.1%)	0.853	19(4.6%)	23(5.5%)	0.436
2	54(12.9%)	81(19.4%)		42(10.1%)	93(22.3%)		77(18.5%)	58(13.9%)		63(15.1%)	72(17.3%)	
3	64(15.3%)	95(22.8%)		44(10.6%)	115(27.6%)		96(23.0%)	63(15.1%)		83(19.9%)	76(18.2%)	
4	42(10.1%)	39(9.4%)		29(7.0%)	52(12.5%)		51(12.2%)	30(7.2%)		46(11.0%)	35(8.4%)	
N stage												
0	7(1.7%)	10(2.4%)	0.942	3(0.7%)	14(3.4%)	0.459	8(1.9%)	9(2.2%)	0.720	8(1.9%)	9(2.2%)	0.979
1	15(3.6%)	20(4.8%)		9(2.2%)	26(6.2%)		20(4.8%)	15(3.6%)		17(4.1%)	18(4.3%)	
2	129(30.9%)	172(41.2%)		97(23.3%)	204(48.9%)		182(43.6%)	119(28.5%)		154(36.9%)	147(35.3%)	
3	30(7.2%)	34(8.2%)		17(4.1%)	47(11.3%)		39(9.4%)	25(6%)		32(7.7%)	32(7.7%)	
Clinical stage												
III	111(26.6%)	171(41.0%)	**0.036**	83(19.9%)	199(47.7%)	0.454	163(39.1%)	119(28.5%)	0.477	135(32.4%)	147(35.3%)	0.134
IVa	40(9.6%)	32(7.7%)		26(6.2%)	46(11.0%)		47(11.3%)	25(6.0%)		44(10.6%)	28(6.7%)	
IVb	30(7.2%)	33(7.9%)		17(4.1%)	46(11.0%)		39(9.4%)	24(5.8%)		32(7.7%)	31(7.4%)	
HGB (M/F) (g/L)												
<120/110	23(5.5%)	14(3.4%)	**0.023**	17(4.1%)	20(4.8%)	**0.038**	24(5.8%)	13(3.1%)	0.599	18(4.3%)	19(4.6%)	0.864
≥120/110	158(37.9%)	222(53.2%)		109(26.1%)	271(65.0%)		225(54.0%)	155(37.1%)		193(46.3%)	187(44.8%)	
ALB (g/L)												
<43	114(27.3%)	141(33.8%)	0.533	89(21.3%)	166(39.8%)	**0.025**	149(35.7%)	106(25.4%)	0.835	132(31.7%)	123(29.5%)	0.304
≥43	67(16.1%)	95(22.8%)		40(9.6%)	122(29.3%)		96(23.0%)	66(15.8%)		75(18.0%)	87(20.8%)	
Treatment mode												
Concurrent	54(12.9%)	70(16.8%)	1.000	37(8.9%)	87(20.9%)	1.000	72(17.3%)	52(12.5%)	0.664	63(15.1%)	61(14.6%)	1.000
Sequential	127(30.5%)	166(39.8%)		89(21.3%)	204(48.9%)		177(42.4%)	116(27.8%)		148(35.5%)	145(34.8%)	
Total platin dose												
≥300 mg/m^2^	111(26.6%)	155(37.2%)	0.411	84(20.1%)	182(43.6%)	0.439	157(37.6%)	109(26.1%)	0.756	131(31.4%)	135(32.4%)	0.477
<300 mg/m^2^	70(16.8%)	81(19.4%)		42(10.1%)	109(16.1%)		92(22.1%)	59(10.1%)		80(19.2%)	71(17.0%)	
Chemo-agent												
Cisplatin	120(28.8%)	153(36.7%)	0.913	83(19.9%)	190(45.6%)	0.991	165(39.6%)	108(25.9%)	0.118	140(33.6%)	133(31.9%)	0.105
Nedaplatin	55(13.2%)	76(18.2%)		39(9.4%)	92(22.1%)		73(17.5%)	58(13.9%)		61(14.6%)	70(16.8%)	
others	6(1.4%)	7(1.7%)		4(1.0%)	9(2.2%)		11(2.6%)	2(0.5%)		10(2.4%)	3(0.7%)	

ECOG PS, Eastern Cooperative Oncology Group performance status; T, tumor; N, nodal; HGB, hemoglobin; ALB, albumin; M, male; F, female; NLR, neutrophil-to-lymphocyte ratio; PLR, platelet-to-lymphocyte ratio; SII, systemic immune inflammation index; SIRI, systemic inflammation response index.

In bold: Statistically significant.

### Prognostic Significance of NLR, PLR, SII, and SIRI

**Figure 2:**
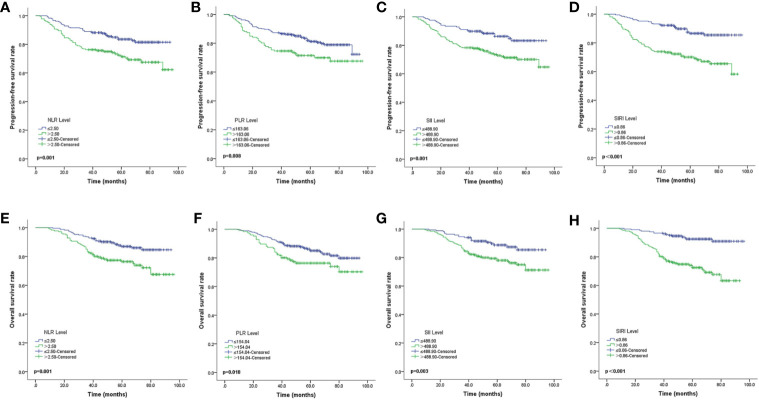
Kaplan-Meier survival curves of locally advanced NPC patients **(A–D)**. Kaplan-Meier curves for PFS according to NLR **(A)**, PLR **(B)**, SII **(C)** and SIRI **(D) (E–H)**. Kaplan-Meier curves for OS according to NLR **(E)**, PLR **(F)**, SII **(G)** and SIRI **(H)**. PFS, progression-free survival; OS, overall survival; NLR, neutrophil-to-lymphocyte ratio; PLR, platelet-to-lymphocyte ratio; SII, systemic immune inflammation index; SIRI, systemic inflammation response index.

The Kaplan-Meier survival curves of PFS with regard to NLR, PLR, SII, and SIRI are shown in **Figures 2A–D**. NLR (p=0.001), PLR (p=0.008), SII (p=0.001), and SIRI (p<0.001) contributed to significantly unfavorable factors of PFS (**Table 3**). Kaplan–Meier OS curves with regard to NLR, PLR, SII, and SIRI are shown in **Figures 2E–H**. NLR (p=0.001), PLR (p=0.018), SII (p=0.003), and SIRI (p<0.001) were significantly correlated with unfavorable OS (**Table 3**). A multivariate Cox regression model including NLR, PLR, SII, and SIRI showed that SIRI was an independent predictor of PFS and OS with HR (hazard ratio) at 2.83 (95% CI 1.561-5.131, p=0.001) and 5.19 (95% CI 2.588-10.406, p <0.001), respectively (**Table 4**).

**Table 3:** Univariate analysis of potential factors associated with PFS and OS.

Variables	PFS			OS		
	Case	MST(m)	*p*	case	MST(m)	*p*
NLR						
High	181	73.97	**0.001**	181	78.87	**0.001**
Low	236	82.91		236	86.03	
PLR						
High	126	73.75	**0.008**	126	79.34	**0.018**
Low	291	81.00		291	84.20	
SII						
High	249	75.6	**0.001**	249	80.67	**0.003**
Low	168	84.6		168	86.80	
SIRI						
High	211	70.37	**<0.001**	211	74.62	**<0.001**
Low	206	87.75		206	91.28	

PFS, progression-free survival; OS, overall survival; NLR, neutrophil-to-lymphocyte ratio; PLR, platelet-to-lymphocyte ratio; SII, systemic immune inflammation index; SIRI, systemic inflammation response index.

In bold: Statistically significant.

**Table 4:** Multivariable Cox regression analyses for PFS and OS.

Variables	PFS		OS	
	HR (95% CI)	*p*	HR (95% CI)	*p*
NLR				
High	0.968(0.564-1.662)	0.906	0.912(0.508-1.640)	0.759
Low				
PLR				
High	1.402(0.868-2.265)	0.167	1.462(0.863-2.479)	0.158
Low				
SII				
High	1.030(0.544-1.951)	0.927	0.766(0.383-1.533)	0.452
Low				
SIRI				
High	2.830(1.561-5.131)	**0.001**	5.190(2.588-10.406)	**<0.001**
Low				

PFS, progression-free survival; OS, overall survival; NLR, neutrophil-to-lymphocyte ratio; PLR, platelet-to-lymphocyte ratio; SII, systemic immune inflammation index; SIRI, systemic inflammation response index; HR, hazard ratio, CI, confidence interval.

In bold: Statistically significant.

## Discussion

NPC is a malignancy with ethnic and geographical distribution preferences (16). Most patients have locally advanced disease with poor prognosis at time of diagnosis. Therefore, there is an urgent need to identify new prognostic factors for NPC patients. In the present study, we evaluated the prognostic value of inflammatory response biomarkers, NLR, PLR, SII, and SIRI in a retrospective cohort of 417 locally advanced NPC patients. The results showed that high NLR, PLR, SII, and SIRI contributed to significantly unfavorable PFS and OS. Furthermore, SIRI was an independent predictor of PFS and OS.

Recent studies have found that inflammation is one of the hallmarks of cancer and is involved in promoting cancer processes. Inflammation associated with cancer development is triggered by a variety of blood immune cells, including neutrophils, macrophages, dendritic cells, and T and B lymphocytes (17). Increasing evidence has shown that systemic immune response markers are promising prognostic biomarkers for various cancers. The combined indicators of neutrophils, lymphocytes, monocytes, and platelets, such as NLR (18, 19), PLR (20), SII (21, 22) and SIRI (23, 24), have been reported to be predictive of cancer prognosis. SIRI is a new systemic inflammatory response biomarker based on neutrophil, monocyte, and lymphocyte counts. Neutrophils aid cancer cells to escape immune surveillance (25). Tumor-associated macrophages, which can promote cancer development and migration, are derived from peripheral monocytes (26). Lymphocytes are major mediators of host anti-cancer immunity. Reduction of peripheral lymphocytes impairs the host’s anticancer immunity and accelerates the progression of cancers. SIRI combines the above cell types and reflects the tumor microenvironment.

The prognostic value of NLR, PLR, and SII has been reported in many types of cancers, including NPC. Pan et al. (27) conducted a retrospective study in stage II NPC patients and found that NLR was an independent prognostic biomarker in stage II NPC patients; NLR≥2.92 was associated with poorer 5-year OS (84.3% vs. 97.4%, *p*=0.001). Another study in NPC patients without distant metastasis reported that NLR≥ 2.28 and PLR≥174 were significantly associated with a shorter OS (*p*<0.05), and NLR≥2.28 was associated with a shorter PFS (*p*<0.05) (28). For NPC patients receiving intensity-modulated radiotherapy, Oei et al. (29) reported that SII was an independent prognostic factor for OS (*p*=0.003), PFS (*p*=0.002), and distant metastasis-free survival (*p*=0.002). Our results were consistent with the above findings, indicating that NLR, PLR, and SII may act as prognostic biomarkers for locally advanced NPC.

SIRI is a novel systemic inflammatory response biomarker that is valuable for predicting the prognosis of cancers. Qi Q et al. (30) conducted a cohort study of patients with advanced pancreatic carcinoma who received palliative chemotherapy, and found that SIRI≥1.8 had a shorter time to progression (HR: 2.348; 95% CI: 1.559-3.535; *p*=0.003) and shorter OS (HR: 2.789; 95% CI: 1.897-4.121; *p <*0.001). In patients with head and neck squamous cell carcinomas, research showed that compared with SIRI<1.1, patients with SIRI between 1.10 and 2.80 had a 1.92 times higher risk of disease-specific death (95% CI: 1.33‐2.82; *p*=0.001), and patients with SIRI >2.80 had a 2.89 times higher risk (95% CI: 1.86‐4.42; *p*=0.0001), SIRI was an independent predictor of disease-specific survival (31). SIRI was also investigated in patients with NPC. In a cohort of 285 patients with NPC, ROC curves showed that the AUC obtained with SIRI was superior to the AUC of other parameters such as NLR or PLR, and SII (*p <*0.001) were significantly associated with PFS and OS (13). These results were consistent with the results of our study, indicating that SIRI acts as a superior prognostic factor for locally advanced NPC patients.

The present study has some inherent limitations. First, it was a single-center retrospective study with a comparatively small sample size, and no validation group was used to confirm the results. Second, the inflammatory response biomarkers NLR, PLR, SII, and SIRI could be affected by various factors such as inflammation and infection, which may have biased the results. Third, the prognosis of patients may be affected by other factors, including EBV-DNA (32), LDH (33), CRP (34), and D-dimer (35). However, in the present study, due to incomplete data, these factors were not considered for evaluation, and this may have biased the results. Finally, the cut-off value for SIRI varied in prior studies, and the optimal cut-off value still needs further investigation. Thus, the results should be interpreted with caution.

In summary, the present study showed that SIRI was an independent predictor of PFS and OS in locally advanced NPC patients. It is a convenient and easy-to-use biomarker that may help in better patient stratification and treatment optimization. Early intervention for system inflammation may be a promising strategy to improve the prognosis of locally advanced NPC patients, warranting further investigation.

## Data Availability Statement

The original contributions presented in the study are included in the article/supplementary material. Further inquiries can be directed to the corresponding author.

## Ethics Statement

The studies involving human participants were reviewed and approved by the ethics committee of the Second Xiangya Hospital, Central South University. Written informed consent to participate in this study was waivered by the ethics committee.

## Author Contributions

TH developed the idea for the study, analyzed and interpreted the patient data, and revised the manuscript. YF collected the patient data and was a major contributor in writing the draft of manuscript. SW, WZ, YH, JAM, and PL collected and cured the data. XL and CH interpreted the data and supervised the research. All authors contributed to the article and approved the submitted version.

## Funding

This research was supported by the Natural Science Foundation of Hunan Province (2020JJ5807).

## Conflict of Interest

The authors declare that the research was conducted in the absence of any commercial or financial relationships that could be construed as a potential conflict of interest.
